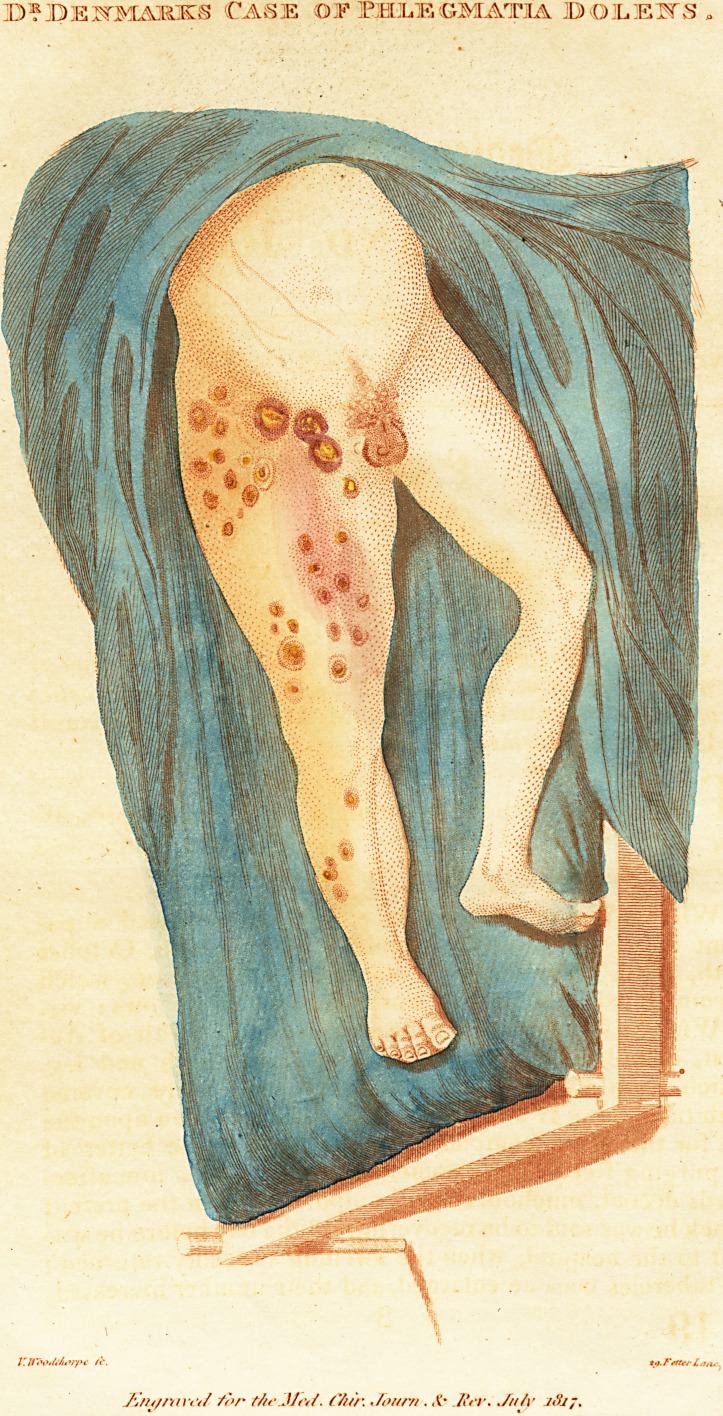# A Case of Phlegmatia Dolens, or a Disease in All Respects Similar, Which Occurred in a Male Subject

**Published:** 1817-07

**Authors:** Alexander Denmark

**Affiliations:** in the Royal Naval Hospital at Haslar


Dr. DENMARK'S CASE OF PHLEGMATIA DOLENS.

V. Woodthorpe fc.

29 Fetter Lane,

*Engraved for the Med. Chir. Journ. & Rev. July 1817.*

THE

### Medico-Chirurgical

### JOURNAL AND REVIEW.

VOL. IV.] JULY, 1817. [NO. 19.

### PART I.

### ORIGINAL COMMUNICATIONS.

*A Case of Phlegmatia Dolens, or a Disease in all respects similar, which occurred in a Male Subject in the Practice of* ALEXANDER DENMARK, M. D. in the Royal Naval Hospital at Haslar.

THIS Case, with the accompanying Sketch, representing the Disease in its worst stage, I have transcribed from my Note-Book, on account of its singularity.

William Tynon, seaman, ætat. 25, was admitted a patient of mine from His Majesty's Ship Achille, October 10th, 1813. The surgeon's statement of the case, which accompanied him to the hospital, was as follows: viz. "Wm. T. was put upon the sick list on the 10th of August, complaining of pain of the right thigh and leg, which were swollen, livid, and here and there covered with tubercles. It was the third time he had been upon the list for the same complaint, which he got the better of by purging freely with calomel and jalap; giving him afterwards decoct. cinchon. et acid. sulph."—and in the present attack he was said to be recovering till the day before he was sent to the hospital, when the swelling suddenly returned; the tubercles became enlarged, and their number increased, some of which the surgeon scarified, when they discharged a thin watery fluid mixed with blood.

Signed, *R. W. Surgeon, Achille.*

On examining the patient, I found the whole of the right extremity much enlarged, tense to the touch, not retaining the impression of the finger, and having a white shining appearance. The surface of the limb, particularly on the inside, was irregularly studded with various sized tubercles, or knobs, from that of a pea to a small walnut; the apices of which resembled hips in colour, more than any thing else that I can compare them to. They had a smooth shining surface too, not unlike that fruit. The inguinal glands were exceedingly enlarged; irregularly knotty; firm, and resisting pressure; and certainly more like an inanimate substance, than that of a living vascular part. They had an elevated, craggy appearance, with deep fossæ; and their apices were not unlike the tubercles already described, but larger. On rubbing the hand over the limb, the sensation communicated was not unlike what one would feel on rubbing it over a hair bottomed chair with brass nails; as, I think, has already been described by some author.

At this time he did not complain of much pain in the limb, unless he made an effort to move it; nor was a considerable degree of pressure capable of inducing any alteration in his sensations. He had a languid, pale, leucophlegmatic countenance; his pulse was rather feeble, but not frequent; appetite variable. Excretions, by perspiration and urine, natural as to quantity; but the latter always deposited a thick white sediment. His intestines were slow, and generally required the aid of brisk purgatives. I could not perceive any thing particular either in his gums or the fœtor of his breath. The energy of his mental faculties appeared to be much below par, evinced by apathy and a dull reserve; indeed, he showed an aversion to every sort of exertion, both mental and corporeal. His own description of his complaint was, consequently, unsatisfactory. He never made any observations without being first interrogated, and then in as laconic a way as possible; both the surrounding scenery and passing events were alike indifferent to him.

He thought his present illness was occasioned by cold. The swelling first began with an indolent enlargement of the glands in the groin, after which the ankle became swollen, (but whether with œdema, was not ascertained); and, lastly, the whole member, with progressive loss of strength and incapacity for locomotion. I ordered the warm bath every third day, with diligent friction and bandaging, accompanied, at first, with hydragogue cathartics, and succeeded by infus. digitalis, sodæ subcarb. and light bitter tonics. Want of success next induced the exhibition of Port wine and animal food; but at the end of a fortnight his general debility was evidently increased. The intestinal canal more torpid. Urine more loaded, sometimes depositing a lateritious sediment, sometimes a white flocculent one. His nights were bad, and his lassitude augmented. The digitalis had been pushed till the stomach became affected, without its having in any visible way altered the renal secretion. I now gave him decoct. cinchon. porter, an additional quantity of wine, and opiates, administering purgatives as occasion required; and warm vinegar fomentations to the limb. For a short time, he seemed to improve under this treatment. The tops of some of the largest of the tubercles in the groin and thigh broke, and discharged a lymphatic fluid. The thigh, at the same time, became softer, and the extremity, about the foot and ankle, changed its tense and elastic feel for one slightly œdematous. It must be observed, by the way, that this breaking of the tubercles, did not lessen the unyielding induration about their bases. It was more like an excoriation in the first instance, occasioned by the hot vinegar, and afterwards turning into an adhering white slough, suffering destruction from impaired vascular action; and although dead, pertinaciously adhering to the subjacent living parts, from a continuation of the same cause; namely, want of action, sufficiently vigorous, to form a line of separation for the expulsion of the sloughs. There now appeared an equably diffused œdematous swelling above the ilium; indeed, extending from the groin over the whole right side of the abdomen, displaying the superficial abdominal and thoracic veins much enlarged. The scrotum and prepuce also partook of the same lymphatic swelling.

On the 12th of November, (one month after his admission into the hospital, and three from the commencement of the disease) a retention of urine came on, requiring the daily introduction of the catheter. A few days afterwards it became incontinent, from an over-distended bladder, he having obstinately refused so frequent an introduction of the catheter. The prepuce and scrotum became excoriated, and a bad sore supervened upon the sacrum, which spread rapidly. He evinced great disinclination towards having any thing done for him, observing that he was sensible of the approach of death, and only desired, (in conformity with his disposition from the first) not to be disturbed. He lingered, however, in this miserable state till the morning of the 10th of December, when he expired.

### Dissection.

On removing the teguments of the thigh, a great quantity of serum oozed out; but whether it had been extravasated in the cellular substance, or issued from the lymphatics divided by the knife, I am unable to say. The cellular membrane under the skin was thickened, and cut hard. The tubercles seemed to rise from the very exterior of the cellular substance, and cut like indurated glands: the skin covering them was not thickened, but merely elevated. Poupart's ligament, which was difficultly discoverable, was raised into an arch, so as to form a segment of a circle, by the immense morbid tumefaction of the parts immediately under it, which filled the whole of the space between the anterior superior spinous process of the ilium and tuberosity of the pubis. This solid inelastic mass I was obliged to cut out in lumps, for there was here no visible distinction of parts, except the femoral artery, vein, and anterior crural nerve. I cut through the tumour upon the site of these vessels by guess, and found that the artery and vein were diminished at least one half in their diameters, and were separated from each other, and also from the crural nerve, to a considerable distance, by the interposition of this diseased substance, which had taken place even within the sheath of the vessels. The fibres of the psoas magnus and iliacus internus, as they pass from the abdomen, were not discoverable, but seemed, together with the different fasciæ, cellular substance, glands, &c. to be blended and identified with this apparently homogeneous substance, to which the great blood vessels of the thigh firmly adhered, as if immoveably imbedded in it. As the pressure diminished below the groin, the muscular fibres and other parts assumed their natural appearance; but the lymphatics and cellular substance in the course of the blood vessels down the thigh partook of the same affection. The dissection of this swelling resembled the cutting of a melon; and the lymphatics within the pelvis and abdomen were, to a certain extent, similarly diseased. In prosecuting the dissection from the groin upwards, the morbid thickening gradually diminished, and ultimately terminated at the last dorsal vertebra, about the beginning of the ductus thoracicus. A transverse incision made over the last lumbar vertebra through the morbid lymphatics and large blood vessels, down to the bone, showed a diseased thickening of more than two inches. On dissecting this from the bone, it was a good deal like the half boiled skin of a calves head, only it felt slippery between the fingers, from the exudation of lymph. There was also a lymphatic tumour opposite and near to the right kidney. The lymphatics within the pelvis in the hollow of the sacrum and round the neck of the bladder, were in like manner affected. The neck of this viscus was completely and thickly encircled, so as materially to impede its functions; and this may, in some measure, account for the ischuria and subsequent enuresis which this poor man laboured under during the last month of his illness.

Never having seen a Case of Phlegmatia Dolens, I am not competent to affirm that this case agrees in all its characters with the symptoms and morbid structure of that disease; but I feel convinced, from all I have read upon the subject, that it more than simulates it; and as it has not yet been described, as far as I know, as having occurred in the male subject, although Dr. Ferriar and others have met with it unconnected with parturition, in females of an advanced age; this case must be held as highly interesting, in a pathological point of view. Dr. Ferriar's opinion then of the disease, "that it may exist independently of every circumstance regarding parturition," so far appears rational; but whether it be owing to lymphatic inflammation, as he says, is not so easily decided. If Mr. White's idea of the proximate cause be correct, "obstruction, detention and accumulation of lymph in the limb, and the lymphatic obstruction to be as high up at least as where they (the lymphatics) enter the pelvis, under Poupart's ligament," how are we to account in the present case for the extension of the disease within the pelvis and abdomen? Mr. Trye thinks also that it is owing to "obstruction of the lymphatic trunks, excited by pressure or the absorption of acrimonious matter." Dr. Denman believes the disease first to arise in the inguinal glands "by the absorption of some irritating principle in the uterine secretions;" but if this were true, one would suppose, that the superficial lymphatic glands in the groin would only become affected, as in the venereal absorption, causing bubo. Dr. Hull supposes the proximate cause to "consist in an inflammatory affection producing suddenly a considerable effusion of serum and coagulable lymph from the exhalents into the cellular membrane of the limb."—As I wish to avoid being led into the fanciful regions of hypothesis, I shall leave the nature of the various phænomena, together with the proximate cause of this disease, for the solution of others more competent to decide.

P. S. The characteristics of this tumefaction being strikingly different from those of œdema, we may infer, that the nature of the effused fluid is also different, by which the permeability of the cellular membrane may be altered, so as to stamp the phlegmatia dolens with a peculiarity of character, and give to it a diagnostic difference wholly distinct from œdema. Obstruction alone then, of the lymphatic trunks, will be inadequate to the peculiar swelling of phlegmatia dolens. Inflammation must, I conceive, be superadded; or, it may, perhaps, be the first link in the chain of morbid action, so as to cause the effusion of coagulable lymph, as well as serum; blocking up the cells, rendering them less pervious, and thereby giving the tense, elastic feel externally to the phleg. dol. totally different from the yielding, plastic tumefaction of anasarca. It will be allowed, that this local lymphatic inflammation and obstruction, may be occasioned by other causes than those of pressure or absorption of morbid secretions; and, according to Dr. Ferriar, give existence to the disease independently of parturition. After the commencement of the complaint, pressure, indeed, occasioned by the indurated inguinal glands may aggravate and render it, in treatment, less tractable. Parturient women, from their greater liability to the predisponent causes, may likewise be considered more obnoxious to the disease. But the pathological view we have taken of the proximate cause, if correct, constitutes it a disease incident to both sexes, and at any age. Have we any dissection of phlegmatia dolens, in parturient women, to prove that the lymphatic trunks within the pelvis and as high as the receptaculum chyli (being a continuation of those of the extremity) are not diseased, as they have appeared to be in the present case? And may not the inflammation, obstruction and effusion, commence in the abdomen instead of below Poupart's ligament, as is usually supposed?

ALEXANDER DENMARK, M. D.

Late Physician to the Mediterranean Fleet.

*Portsmouth, May,* 1817.

## Figures and Tables

**Figure f1:**